# Competition Between Butyrate Fermenters and Chain-Elongating Bacteria Limits the Efficiency of Medium-Chain Carboxylate Production

**DOI:** 10.3389/fmicb.2020.00336

**Published:** 2020-03-06

**Authors:** Bin Liu, Sabine Kleinsteuber, Florian Centler, Hauke Harms, Heike Sträuber

**Affiliations:** Department of Environmental Microbiology, Helmholtz Centre for Environmental Research – UFZ, Leipzig, Germany

**Keywords:** carboxylate platform, reactor microbiota, anaerobic fermentation, mixed culture, lactate-based chain elongation, ecological interactions, lactic acid bacteria

## Abstract

Medium-chain carboxylates such as *n*-caproate and *n*-caprylate are valuable chemicals, which can be produced from renewable feedstock by anaerobic fermentation and lactate-based microbial chain elongation. Acidogenic microbiota involved in lactate-based chain elongation and their interplay with lactic acid bacteria have not been characterized in detail yet. Here, the metabolic and community dynamics were studied in a continuous bioreactor with xylan and lactate as sole carbon sources. Four succession stages were observed during 148 days of operation. After an adaptation period of 36 days, a relatively stable period of 28 days (stage I) was reached with *n-*butyrate, *n-*caproate and *n-*caprylate productivities of 7.2, 8.2 and 1.8 gCOD L^–1^ d^–1^, respectively. After a transition period, the process changed to another period (stage II), during which 46% more *n-*butyrate, 51% less *n-*caproate and 67% less *n-*caprylate were produced. Co-occurrence networks of species based on 16S rRNA amplicon sequences and correlations with process parameters were analyzed to infer ecological interactions and potential metabolic functions. Diverse functions including hydrolysis of xylan, primary fermentation of xylose to acids (e.g., to acetate by *Syntrophococcus*, to *n-*butyrate by *Lachnospiraceae*, and to lactate by *Lactobacillus*) and chain-elongation with lactate (by *Ruminiclostridium* 5 and *Pseudoramibacter*) were inferred from the metabolic network. In stage I, the sub-network characterized by strongest positive correlations was mainly related to the production of *n-*caproate and *n-*caprylate. Lactic acid bacteria of the genus *Olsenella* co-occurred with potentially chain-elongating bacteria of the genus *Pseudoramibacter*, and their abundance was positively correlated with *n-*caproate and *n-*caprylate concentrations. A new sub-network appeared in stage II, which was mainly related to *n-*butyrate production and revealed a network of different lactic acid bacteria (*Bifidobacterium*) and potential *n-*butyrate producers (*Clostridium sensu stricto* 12). The synergy effects between lactate-producing and lactate-consuming bacteria constitute a division of labor cooperation of mutual benefit. Besides cooperation, competition between different taxa determined the bacterial community assembly over the four succession stages in this resource-limited system. During long-term reactor operation under constant conditions, chain-elongating bacteria were outcompeted by butyrate-producing bacteria, leading to the increase of *n-*butyrate yield at the cost of medium-chain carboxylate yields in this closed model system.

## Introduction

The production of platform chemicals and fuels from renewable resources is a major focus of a circular economy. The carboxylate platform offers the opportunity to sustainably produce bio-based chemicals such as medium-chain carboxylates (MCCs), which are mainly produced from coconut and palm kernel oils (Anneken et al., 2006). MCCs can be widely utilized in agriculture and industry, for example, as precursors for the production of fragrances (Kenealy et al., 1995), antimicrobial agents (Desbois, 2012) and drop-in biofuels (Urban et al., 2017). Besides the multi-functional applications, MCC production in a biorefinery context also meets the requirement of sustainable development because it replaces fossil resources and botanical oils such as palm kernel oil.

Medium-chain carboxylates are monocarboxylates that contain six to twelve carbon atoms. In this study, we subsume their dissociated and undissociated forms as carboxylates, with a main focus on *n*-caproate (C6) and *n*-caprylate (C8). In a process known as chain elongation (CE), intermediates of acidogenesis such as acetate (C2) and *n*-butyrate (C4) can be elongated to MCCs by adding acetyl-CoA in reverse β-oxidation cycles (Spirito et al., 2014). C2 or C4 need to be transformed to acetyl-CoA or butyryl-CoA, respectively, as initial substrate for elongation in the reverse β-oxidation. Thioesterase or coenzyme A transferase can be used as terminal enzymes for MCC production. Ethanol has been well described as electron donor providing energy for coupling acetyl-CoA formation and elongating acyl-CoA units (Seedorf et al., 2008). Besides ethanol, lactate also suits as electron donor for the CE process (Zhu et al., 2015; Kucek et al., 2016; Khor et al., 2017). Feedstocks that are rich in lactate (e.g., ensiled plant biomass) or lactate-precursors (e.g., carbohydrates) are thus promising substrates for the production of MCCs.

Phylogenetically different species have been described as CE bacteria that can produce C6 and even C8. The genera *Clostridium* (*C. kluyveri*), *Eubacterium* (*E. limosum* and *E. pyruvativorans*), *Megasphaera* (*M. elsdenii*, *M. indica*, and *M. hexanoica*), and *Caproiciproducens* (*C. galactitolivorans*) all include chain-elongators (Angenent et al., 2016). The recent discovery of chain-elongating *Ruminococcaceae* bacterium CPB6 (Zhu et al., 2017) suggests that further C6-producers remain to be discovered. To convert complex organic substrates (e.g., corn silage), the joint efforts of different trophic groups in a food web are required. However, the substrate spectrum of pure strains that are able to carry out CE is limited. Therefore, multi-species reactor microbiota can be considered more viable for the utilization of complex substrates due to their broad metabolic capacity. On the one hand, diverse functional groups in the microbial community may cooperate in metabolizing complex substrates like polysaccharides. Recent studies suggested that lactic acid bacteria (LAB) play an important role in lactate-based CE (Andersen et al., 2017; Scarborough et al., 2018b; Lambrecht et al., 2019). However, in such open-culture reactor systems, other intermediates including ethanol may also be produced from hydrolysis and acidogenesis of complex substrates, making it hard to discern the role of lactate. On the other hand, bacterial competition cannot be avoided in a resource-limited bioreactor. From an ecological perspective, the relationships between LAB and CE bacteria are still unexplored.

Aforementioned ecological interactions have been commonly investigated in other engineered microbial ecosystems, such as activated sludge of wastewater treatment plants (Ju and Zhang, 2015) and anaerobic digesters (Ziels et al., 2018). For chain elongation systems, it is not clear how cooperation and competition influence the process performance and shape the structure of the microbial community. To address this question, we studied lactate-based CE in a simplified lab-scale system. To reduce the complexity of a real system such as the anaerobic fermentation of ensiled plant biomass, we applied a model system with sterilized mineral medium containing xylan and lactate as sole carbon sources. We hypothesized that lactate formed *in situ* by sugar fermentation can be converted to MCCs in the CE process. By monitoring the process performance during long-term operation under constant conditions and by investigating the microbial community structure based on 16S rRNA amplicon sequencing, we intended to understand how the community dynamics affects the MCC productivity in our system. By performing network analysis, we aimed to elucidate the ecological interactions between the different functional groups LAB and CE bacteria.

## Materials and Methods

### Growth Medium and Inoculum

The basal medium was modified from a previous study in which lactate was used to produce *n-*caproate (Weimer and Moen, 2013). It contained per liter: 0.054 g MgCl_2_ ⋅ 6H_2_O, 0.065 g CaCl_2_ ⋅ 2H_2_O, 1.612 g NH_4_Cl, 5.470 g KH_2_PO_4_, 10.415 g K_2_HPO_4_, 0.032 g Na_2_CO_3_, 0.030 g cysteine-HCl, 0.5 g yeast extract, 1 mL of vitamin solution (biotin 20 mg/L, folic acid 20 mg/L, pyridoxine 100 mg/L, thiamine 50 mg/L, riboflavin 50 mg/L, nicotinic acid 50 mg/L, calcium pantothenate 50 mg/L, vitamin B_12_ 20 mg/L, *p*-amino benzoic acid 80 mg/L, lipoic acid 50 mg/L), and 1 mL of trace element solution (FeCl_2_ ⋅ 4H_2_O 1.5 g/L, CuCl_2_ ⋅ 2H_2_O 2 mg/L, CoCl_2_ ⋅ 6H_2_O 190 mg/L, MnCl_2_ 100 mg/L, Na_2_MoO_4_ ⋅ 2H_2_O 36 mg/L, NiCl_2_ ⋅ 6H_2_O 24 mg/L, Na_2_WO_4_ ⋅ 2H_2_O 20 mg/L, Na_2_SeO_3_ ⋅ 5H_2_O 3 mg/L, ZnCl_2_ 70 mg/L, H_3_BO_3_ 6 mg/L). The medium was adjusted with 1 M NaOH solution to the operating value of pH 5.5. Lactate and xylan were fed daily as carbon sources to the reactor.

The inoculum was taken from a lab-scale CE reactor fed with lactate-rich corn silage (Lambrecht et al., 2019). This semi-continuous stirred tank reactor with a working volume of 12 L and operated at pH 5.5 had been daily fed with 3 L substrate mix resulting in a substrate retention time of 4 days. The bioreactor microbiota showed a stable performance for producing MCCs. Initially, 1 L fermentation broth taken from the lab-scale CE reactor was sieved (mesh size 2 mm) to remove particles of the corn silage. After the filtration, 875 mL liquid phase was used as inoculum and pumped into the reactor flushed with nitrogen. No chemical agent for the specific inhibition of methanogenesis was applied.

### Bioreactor Operation and Sampling

A BioStat-A plus bioreactor (Sartorius AG, Göttingen, Germany) with 1 L working volume was used. The tank reactor was operated at 38 ± 1°C and at a constant stirring rate of 150 rpm. The pH was automatically controlled at 5.5 by addition of 1 M sodium hydroxide solution. For the daily feeding, 1.47 g lactic acid (85%, FCC grade; Sigma Aldrich, St. Louis, MI, United States) diluted in 50 mL deionized water, and 1.25 g water-soluble xylan (more than 95% xylooligosaccharides, from corncob; Roth, Karlsruhe, Germany) dissolved in 75 mL medium were supplied. Once a day, 125 mL effluent was taken before feeding corresponding to a hydraulic retention time (HRT) of 8 d. A gas-tight bag (produced on-site using thermoplastic coated aluminum foil) was used to collect the produced gas or for compensating underpressure in the reactor system. It was connected after a MilliGascounter (MGC-1; Ritter, Bochum, Germany). A buffer bottle was installed between the MGC and the bioreactor preventing the sealing fluid of the MGC-1 to be sucked into the reactor in case of underpressure. A septum was placed in the gas pipe for gas sampling. Gas samples of 1 mL were taken with a syringe flushed with nitrogen and injected into 20-mL gas-tight glass vials that had been flushed with argon for 20 min.

Reactor effluent was used for cell concentration measurement. For other analyses, liquid samples were collected twice per week and centrifuged for 10 min at 20,817 × *g* (Centrifuge 5417R; Eppendorf, Hamburg, Germany). The supernatants were used for measuring concentrations of xylan, total ammonia nitrogen (TAN), carboxylates and alcohols. Pelleted biomass samples from 50 mL reactor effluent were washed three times with phosphate buffer (PBS, 1.8 g L^–1^ Na_2_HPO_4_, 0.223 g L^–1^ NaH_2_PO_4_, 8.5 g L^–1^ NaCl in deionized H_2_O pH 7.2; centrifugation at 10,000 × *g*, 10 min, 10°C) before determination of the cell dry weight. For microbial community analysis, the pelleted cells from 2 mL samples were washed with 100 mM Tris–HCl buffer pH 8.5 and stored at −20°C.

### Analyses of Process Parameters

Daily gas production was monitored using MGC-1 and normalized to standard pressure and temperature conditions (101.325 kPa and 273.15 K) as described by Sträuber et al. (2018). Gas composition was analyzed in triplicate for H_2_, CO_2_, N_2_, O_2_, and CH_4_ by GC according to Urban et al. (2017).

The TAN concentration was monitored twice a week as described previously (Popp et al., 2015).

Concentrations of carboxylates and alcohols were determined by gas chromatography (GC) in triplicate after derivatization of the analytes as previously reported (Urban et al., 2017). Here, 1 mL of 2-ethylbutyric acid was used as the internal standard. For the derivatization, 0.5 mL methanol and 2.5 mL 1 M sulfuric acid were added. Xylan was measured with a modified classical dinitrosalicylic acid reagent method (Miller, 1959). Xylan in the supernatant was acidified with 1 M sulfuric acid and then hydrolyzed at 121°C for 60 min. Before using the reagent, the pH of the hydrolysate was adjusted to be neutral.

The cell mass concentration was determined by measuring the optical density (OD) at 600 nm (spectrophotometer Genesys 10 S, Thermo Scientific Inc., Waltham, MA, United States) and correlated with the cell dry mass. For determining the cell dry mass, the cell pellets were dried at 60°C for 48 h before weighing (six replicates). Considering the microbial community shifts, we calculated a mean correlation coefficient (1 OD_600_ = 0.581 g L^–1^) based on all cell dry mass measurements except the first measurement (Supplementary Material B). The chemical oxygen demand (COD) of microbial biomass was measured with a COD kit (LCK 714, Hach Lange GmbH, Germany) as described by Bonk et al. (2018).

The electron recovery was calculated according to the Eqs 1 and 2.

(1) $$\eta_{\text{e}\,1^{-}}=\frac{{\text{q}}_{\text{C4}-\text{C10}}\,}{{\text{q}}_{\text{l}\,\text{a}\,\text{c}}\,+\,{\text{q}}_{\text{x}\,\text{y}\,\text{l}\,\text{a}\,\text{n}}}\,100\%$$

(2) $$\eta_{\text{e}\,2^{-}}=\frac{{\text{q}}_{\text{biomass}}}{{\text{q}}_{\text{l}\,\text{a}\,\text{c}}\,+\,{\text{q}}_{\text{x}\,\text{y}\,\text{l}\,\text{a}\,\text{n}}}\,100\%$$

Where η_*e1–*_ and η_*e2–*_ are defined as electron recovery, *q*_*C*4−*C*10_is the sum of all electrons in the carboxylates (*iso-*butyrate, *n-*butyrate, *iso-*valerate, *n-*valerate, *n-*caproate, *n-*heptanoate, *n-*caprylate, *n-*nonanoate, *n-*decanoate), *q*_biomass_ is the number of electrons in the cell biomass, *q*_lac_ and *q*_xylan_ are the numbers of electrons of the input substrates lactate and xylan.

### Microbial Community Analysis

Genomic DNA was extracted from frozen pellets using the NucleoSpin Microbial DNA Kit (Macherey-Nagel, Germany) according to the instructions of the manufacturer. Methods for DNA quantification and quality control were as described before (Lucas et al., 2015). The community dynamics throughout the experiment was studied by terminal restriction fragment length polymorphism (T-RFLP) fingerprinting. For this purpose, bacterial 16S ribosomal RNA (rRNA) genes were amplified by polymerase chain reaction (PCR) using the MyTaq^TM^Mix (Bioline, Germany) and the primers 27f (labeled with phosphoramidite fluorochrome 5-carboxyfluorescein (FAM); 5′-GAG TTT GAT CMT GGY TCA G-3′) and 1492r (5′-TAC GGY TAC CTT GTT ACG ACT T-3′) (according to Lane, 1991). With a total volume of 12.5 μL, the mixtures of PCR reaction contained 6.25 μL of MyTaq^TM^ Mix, 3.85 μL of nuclease free water, 0.7 μL of each primer (5 pmol) and 1 μL sample DNA (diluted to 20 ng μL^–1^). The cycling protocol included an initial denaturation at 95°C for 1 min, followed by 30 cycles of denaturation at 95°C for 15 s, primer annealing at 58°C for 15 s, elongation at 72°C for 10 s, and a final elongation step at 72°C for 15 min. Amplicons were purified using the SureClean Kit (Bioline, Germany) and quantified using a NanoDrop ND 1000 spectral photometer (Thermo Fisher Scientific, United States). For T-RFLP analysis, 80 ng of 16S rRNA amplicons were digested over night at 37°C with 2 U of restriction endonuclease *Msp*I or *Rsa*I (New England Biolabs, Germany). The MapMarker1000 (BioVentures Inc., United States) was applied as fragment size standard. By using capillary electrophoresis with an automatic sequencer (ABI PRISM 3130xl Genetic Analyzer; Applied Biosystems, United States), the terminal restriction fragments (T-RFs) were separated. The electropherograms were analyzed by using the GeneMapper 5 software (Applied Biosystems) and processed by using a script according to Abdo et al. (2006) implemented in R Studio (Version 1.0.143). Low-signal peaks were removed below a threshold of seven times the standard deviation of data sets. T-RFs in the range of 50–1000 bp were included in further analyses.

For analyzing the community composition based on 16S rRNA gene sequences, 25 sampling points representing the different process stages were selected. Amplicon sequencing of 16S rRNA gene fragments was performed on the Illumina MiSeq platform (V3-V4 regions, 2 × 300 bp). The primers 341f (CCT ACG GGN GGC WGC AG) and 785r (GAC TAC HVG GGT ATC TAA KCC) were used for amplification. De-multiplexed sequence data were processed with QIIME2 v2019.1 (Bolyen et al., 2019). Filtering of phiX reads, denoising, merging of paired ends, trimming and chimera detection were done with the plugin of Divisive Amplicon Denoising Algorithm DADA2 (Callahan et al., 2016). The following parameters were used in DADA2: p-trim-left-f 0, p-trim-left-r 0, p-trunc-len-f 250, p-trunc-len-r 200. These were selected by reviewing the Interactive Quality Plot for removing low quality regions of the sequences. Other parameters were used by default. The generated feature table indicates the frequency each amplicon sequence variant (ASV) is observed in each sample. Taxonomic assignment was carried out using a naïve Bayes classifier trained on 16S rRNA gene sequences of the latest Midas database 2.1 (McIlroy et al., 2015). The feature table was rarefied down to the lowest read number (21,214 sequences) for further analyses. Two samples (days 46 and 116) were excluded due to lower read numbers. The de-multiplexed sequence dataset of 25 samples was deposited to the EMBL-EBI database under accession number PRJEB34417.

### Statistical Analyses

Non-metric multidimensional scaling (NMDS) was used as ordination technique for dissimilarity matrices based on the T-RFLP profiles including occurrence and relative abundance of terminal restriction fragments (T-RFs). The Bray-Curtis dissimilarity index was used to evaluate the dynamics of the microbial communities (Bray and Curtis, 1957) reflected by the distances between data points. Smaller distances indicate higher similarities of community compositions. Based on the “vegan” R package (Oksanen et al., 2016), the “envfit” algorithm was used to calculate the relationships between abiotic parameters and T-RFLP profiles. The significance threshold was set to 0.01, which was tested by Monto Carlo test with 999 permutations.

Alpha diversity based on T-RFLP and ASV data was evaluated by using the ecological indices including richness, diversity and evenness as described by Lucas et al. (2017). Diversity of order one (D1) and evenness of order one (E1) quantify the diversity and evenness by weighting all present types equally, whereas diversity of order two (D2) and evenness of order two (E2) give more weight to the dominant types than to the rare types.

Significant differences of mean carboxylate recoveries were tested by Student’s *t*-test (^∗∗∗^*P* < 0.001, ^∗∗^*P* < 0.01, and ^∗^*P* < 0.05, *n* = 6). Significant differences of mean biomass recoveries were tested by Mann-Whitney rank sum test (^∗∗∗^*P* < 0.001, ^∗∗^*P* < 0.01, and ^∗^*P* < 0.05, *n* = 6).

Co-occurrence networks based on 16S rRNA amplicon sequence data and abiotic parameters were inferred by following the protocol of Faust et al. (2015), using the CoNet App (v 1.1.1 beta) (Faust and Raes, 2016). Only ASVs that had > 0.1% relative abundance in more than three samples were included in the analysis to reduce spurious correlations. Relative ASV abundances were converted into absolute mean abundances based on total cell concentrations (gram dry mass per liter). Correlations between ASVs and process parameters (time; biomass; concentrations of C2, C4, C6, C8, and lactate; CO_2_ and H_2_ content; gas amount) were also considered in the network. Pearson, Spearman, and Kendall correlation coefficients were computed and if at least one method featured a coefficient below −0.75 or above 0.75, an edge connecting the corresponding ASVs or abiotic parameters was added to the network. All networks were visualized and analyzed for topological features in Cytoscape software (v 3.7.1) (Shannon et al., 2003).

## Results

### Metabolic and Microbial Community Dynamics Over Different Succession Stages

**FIGURE 1:**
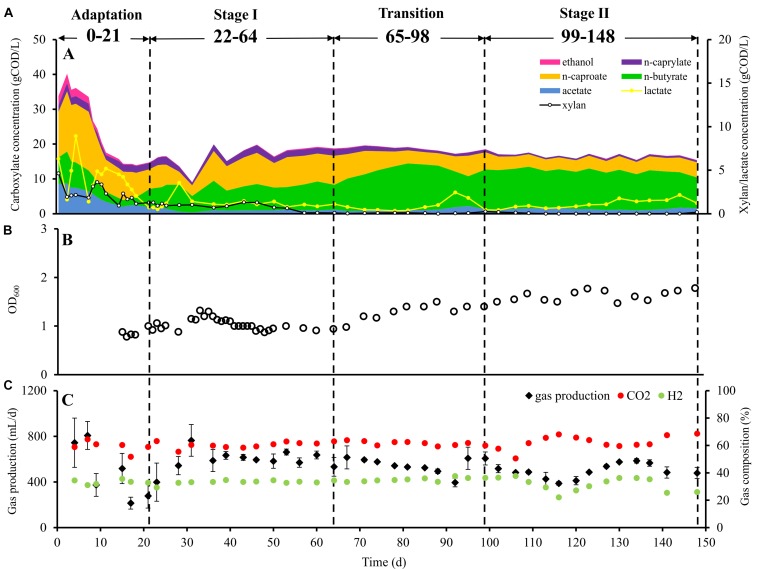
Performance of bioreactor. **(A)** Substrate and product concentrations in the bioreactor during the four succession stages, **(B)** cell concentration (OD_600_ value) in the bioreactor during the four succession stages, and **(C)** daily gas production and gas composition during the four succession stages. Data points for gas production indicate mean values of 3 days. Within 95% confidence intervals, the error bars represent the standard deviation.

**FIGURE 2:**
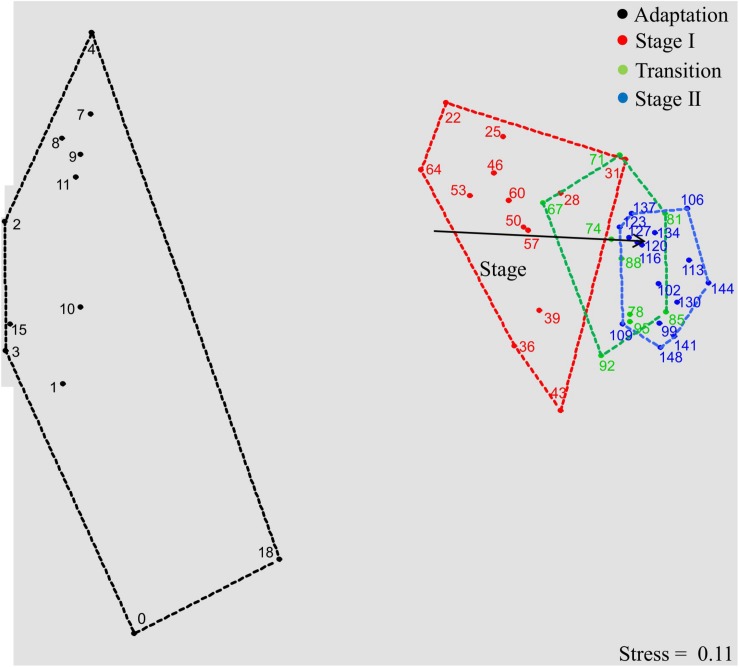
Bacterial community dynamics in the four succession stages, illustrated by a non-metric multidimensional scaling (NMDS) plot of T-RFLP profiles (16S rRNA gene amplicons, restriction enzyme *Rsa*I; the plot based on *Msp*I is shown in Supplementary Figure S1). Data points are named according to sampling days. Proximity of data points represents community similarity based on the Bray-Curtis index. Colored polygons indicate sampling days of each process stage. The vector shows community shifts within the temporal dynamics (*P* < 0.01, significance calculated by Monte-Carlo test with 999 permutations).

**FIGURE 3:**
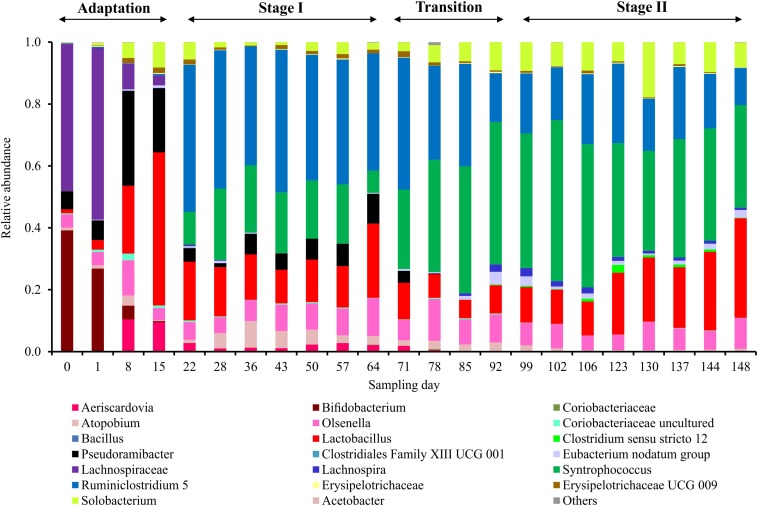
Microbial community composition profiles in the four succession stages based on amplicon sequencing of 16S rRNA genes. Taxonomic classification of amplicon sequence variants (ASVs) was categorized at the genus level. ASVs with relative abundance lower than 0.1% are classified into “Others.”

The microbial chain elongation system analyzed in this study was designed to include hydrolysis and primary fermentation. Xylan and lactate were fed daily over a period of 148 days, and the reactor microbiota produced mainly *n-*butyrate, *n-*caproate and *n-*caprylate (Figure 1A). The gas was composed mainly of carbon dioxide and hydrogen (Figure 1C), with traces of nitrogen and oxygen. No methane was detected in the reactor headspace. The community dynamics analyzed by T-RFLP fingerprinting is shown as NMDS plots (Figure 2 for *Rsa*I and Supplementary Figure S1 for *Msp*I). From 25 samples analyzed by amplicon sequencing, in total 909,240 sequence reads were obtained, which were assigned to 95 ASVs from high-quality sequence reads. Overall, ASVs were affiliated to three phyla (*Firmicutes*, *Actinobacteria* and *Proteobacteria*), six classes (*Clostridia*, *Coriobacteriia*, *Actinobacteria*, *Erysipelotrichia*, *Bacilli* and *Alphaproteobacteria*), seven orders, 11 families and 20 genera with at least 0.1% relative abundance for each ASV (Figure 3). During this long-term reactor operation, four succession stages – adaptation (days 0–21), stage I (days 22–64), transition (days 65–98) and stage II (days 99–148) – were identified based on carboxylate concentration profiles (Figure 1A) and T-RFLP profiles (Figure 2 and Supplementary Figure S1).

In the adaptation stage, a certain share of the carboxylates produced still originated from the liquid inoculum. Large variations in daily gas production and carbon dioxide content were observed (Figure 1C). The T-RFLP profiles (Figure 2) indicate considerable community shifts from the adaptation stage to the formation of a stable community composition (Figure 3). In the adaptation stage, the microbial community was dominated by ASVs assigned to unclassified *Lachnospiraceae*, *Lactobacillus*, *Bifidobacterium*, *Pseudoramibacter*, *Olsenella*, *Aeriscardovia*, *Solobacterium*, and *Atopobium* (Figure 3). Hereafter, it took around 2.5 HRTs for the microbiota to adapt to the given process conditions.

After the adaptation, the dominating genera were distinctly different from those in the inoculum, which indicates the highly selective conditions of our reactor system. In contrast to the strong community shifts during adaptation, data points standing for stage I (red), transition (green) and stage II (blue) are less scattered in the NMDS plot, displaying a relatively lower dissimilarity of the community structures within these periods (Figure 2). After the transition stage, the microbial community shifted from stage I to stage II as indicated by the vector “Stage.” Similar results were obtained by T-RFLP analysis with *Msp*I (Supplementary Figure S1).

Alpha diversity metrics shows that richness, diversity of order one (D1) and as well as evenness of order one (E1) based on the T-RFLP data varied more over time than the respective indices based on the ASVs (Supplementary Figure S2). This could be due to the limitation of the T-RFLP method, which fails to detect rare sequence types. When focusing on the dominant types (D2 and E2), we observed a clear trend that the diversity (D2) was lower in stage II (mean values; ASV: 4.6, *Rsa*I: 2.5 and *Msp*I: 3.5) compared with stage I (mean values; ASV: 6.1, *Rsa*I: 4.7 and *Msp*I: 5.8). The community in stage II was also less even than that in stage I, as E2 was lower in stage II (mean values; ASV: 0.2, *Rsa*I: 0.2 and *Msp*I: 0.2) compared with stage I (mean values; ASV: 0.3, *Rsa*I: 0.4, and *Msp*I: 0.4).

In stage I, the first period with constant carboxylate production over days 46–64 was observed (Figure 1A). With a loading rate of 10.7 gCOD L^–1^ d^–1^ as lactate and 12.1 gCOD L^–1^ d^–1^ as xylan, mean concentrations of 1.0 ± 0.1 gCOD L^–1^ acetate, 7.2 ± 0.7 gCOD L^–1^ *n-*butyrate, 8.2 ± 0.7 gCOD L^–1^ *n-*caproate and 1.8 ± 0.2 gCOD L^–1^ *n-*caprylate were obtained. Additionally, 1.0 ± 0.2 gCOD L^–1^ of lactate and 0.5 ± 0.5 gCOD L^–1^ of xylan were detected during this period. The mean daily gas production was 599.4 ± 85.9 mL d^–1^ in stage I. The gas consisted mainly of CO_2_ (60.4 ± 1.7%) and H_2_ (33.2 ± 0.7%). After the inoculation, still some particles from the seed sludge were retained in the bioreactor, which highly influenced the measured OD values. Therefore, OD values are only shown from day 15 onward (Figure 1B) as we assumed that most of the residual particles in the inoculum were washed out by then. The mean cell mass concentration was 0.66 ± 0.02 g dry mass L^–1^. Here, ASVs assigned to *Syntrophococcus* (17.2 ± 5.9%), *Lactobacillus* (15.9 ± 4.3%), *Pseudoramibacter* (5.9 ± 2.6%), *Olsenella* (7.9 ± 2.4%), *Aeriscardovia* (1.9 ± 0.7%), *Solobacterium* (2.6 ± 1.7%), *Atopobium* (4.3 ± 2.5%), uncultured *Coriobacteriaceae* (0.3 ± 0.2%), *Erysipelotrichaceae* UCG 009 (0.9 ± 0.6%), *Eubacterium nodatum* group (0.3 ± 0.2%), *Lachnospira* (0.1 ± 0.1%), unclassified *Erysipelotrichaceae* (0.1 ± 0.1%), and *Ruminiclostridium* 5 (42.3 ± 3.8%) (mean relative abundance ± standard deviation, *n* = 7) predominated, while ASVs identified as *Bifidobacterium* and unclassified *Lachnospiraceae* were detected below 0.1% of relative abundance. ASVs assigned to *Acetobacter* and *Clostridiales* family XIII UCG 001 were below the detection limit.

From the beginning of the transition interval on day 65, a trend of more C4 production and less C6/C8 production was observed. The daily gas production was not as stable as before. Here, ASVs assigned to *Aeriscardovia* and *Pseudoramibacter* disappeared and *Clostridium sensu stricto* 12 (0.9 ± 0.7%; *n* = 4) emerged (Figure 3).

After the transition period, 46% more C4, 51% less C6 and 67% less C8 were produced compared with stage I. In stage II, we obtained mean concentrations of 1.3 ± 0.3 gCOD L^–1^ acetate, 10.5 ± 1.0 gCOD L^–1^ *n-*butyrate, 4.0 ± 0.3 gCOD L^–1^ *n-*caproate, and 0.6 ± 0.1 gCOD L^–1^ *n-*caprylate. The mean concentration of lactate was 1.6 ± 0.3 gCOD L^–1^, whereas no xylan was detected in stage II. For comparing with the results of stage I, concentrations over days 130–148 were used for calculating mean concentrations over the last six sampling points in each stage. Remarkably, no propionate was detected since stage I. The daily gas production was 21.1% lower than in stage I, with an average of 473.0 ± 84.3 mL d^–1^. The contents of CO_2_ and H_2_ were 62.6 ± 5.0 and 31.3 ± 5.3%, respectively. The fluctuation of the H_2_ content was always consistent with the daily gas production. For CO_2_, the trend was in the reverse direction throughout stage II. The mean daily hydrogen production was 152.3 ± 48.3 mL d^–1^, the mean daily carbon dioxide production was 296.2 ± 48.4 mL d^–1^. Noteworthy, occasional underpressure in stage II was indicated by the sealing fluid of the MGC sucked into the tube toward the reactor. The mean cell mass concentration increased by 42% in stage II up to 0.93 ± 0.07 g dry mass L^–1^. In stage II, ASVs assigned to *Eubacterium nodatum* group, *Lachnospira*, *Lactobacillus*, *Syntrophococcus* and *Solobacterium* increased in their relative abundance to 1.6 ± 0.8, 1.4 ± 0.7, 18.9 ± 7.6, 39.8 ± 6.9, and 9.4 ± 3.6% (stage II, *n* = 8), respectively. ASVs of *Atopobium*, *Ruminiclostridium* 5 and *Olsenella* dropped down to abundances of 0.7 ± 0.5, 19.3 ± 4.4, and 7.2 ± 1.9%, respectively. All uncertainties are represented by 95% confidence intervals.

### Electron and COD Balances in Stage I and Stage II

The electron recovery indicates the partitioning of electrons from electron donors (xylan and lactate) to acceptors as a result of anabolic and catabolic processes. Taking all compounds analyzed by GC into account and considering a total input of 2.85 mol L^–1^ electron equivalents, the electron balances were similar in stage I (92 ± 3%) and stage II (89 ± 4%) (Supplementary Table S1). Comparable results were obtained for the COD balances (Supplementary Table S2). With an input of 23.48 gCOD L^–1^, the COD balances in stage I and stage II were 91.2 ± 0.6 and 92.2 ± 0.8%, respectively.

**FIGURE 4:**
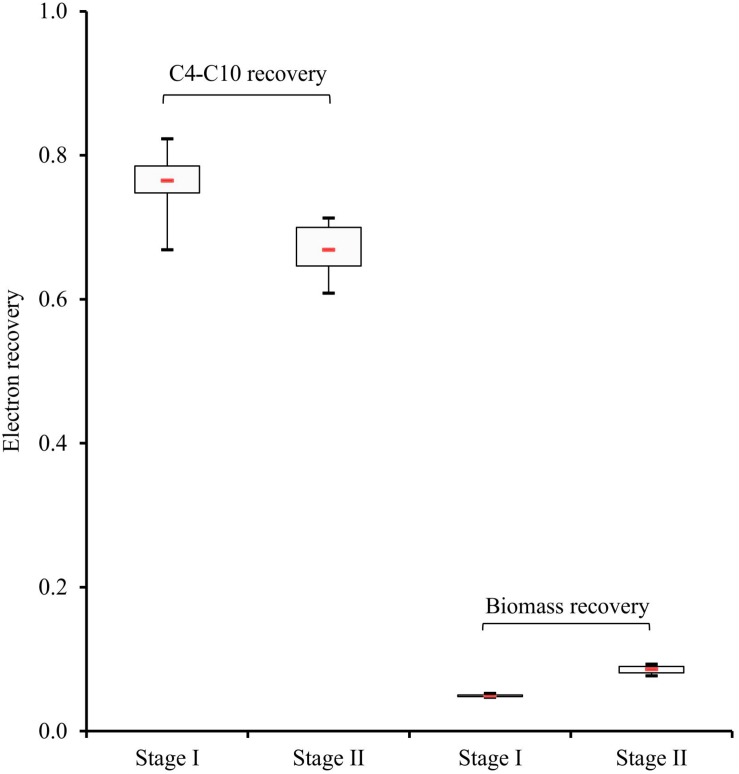
Electron recoveries in carboxylates (C4-C10) and cell biomass, represented by 95% confidence intervals. Boxes indicate the interquartile range between the 25th and 75th percentiles, respectively, the red line inside indicates the median value, mean values between both stages show significant differences (*P* < 0.05).

Most of the electrons were recovered in the C4-C10 products in both stages (Supplementary Table S1 and Figure 4). As shown in Figure 4, the electron recovery (median values) in these CE products decreased by 12% from stage I to stage II as time progressed, which represents a significant difference (two-tailed *P*-value = 0.007). Moreover, compared with stage II, 80% more of the electrons (median values) from consumed xylan and lactate were channeled to cell biomass in stage II, which also shows a significant difference between the two stages (two-tailed *P*-value = 0.002). Thus, electron balances indicate that a higher percentage of substrate was directed to cell biomass synthesis and other non-target CE products with progressing operating time.

### Network Inference in Stage I and Stage II

We constructed three separate networks to analyze the relationships among microbial taxa or process parameters with the aim to reveal potential functions and ecological interactions within the microbial community in our CE reactor.

**FIGURE 5:**
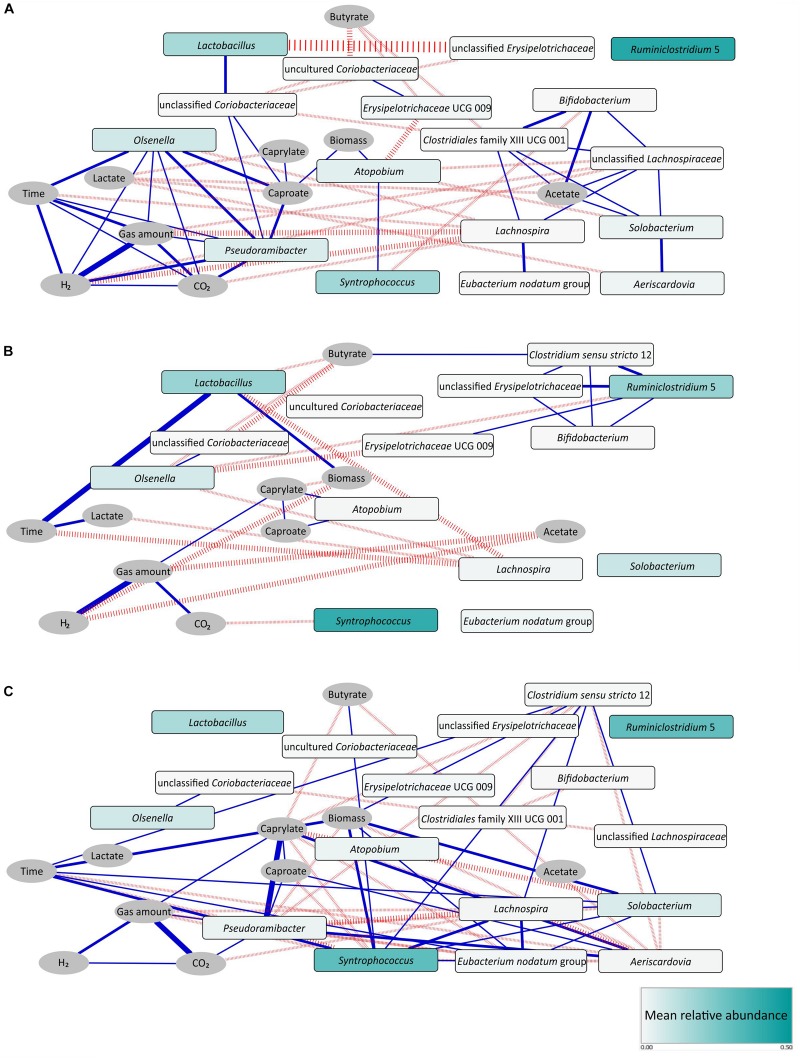
Correlation networks for stage I **(A)**, stage II **(B)**, and stage I - transition stage - stage II **(C)**. Pearson, Spearman, and Kendall correlation coefficients were computed for each pair of microbial taxa (rectangular nodes) and abiotic parameters (elliptic nodes) over the respective period using absolute taxon abundances. Edges indicate a coefficient > 0.75 for positive correlations (blue edges) and < –0.75 for negative correlations (red edges). Edge thickness indicates the number of methods supporting the correlation (1 to 3). Shading of the microbial taxon nodes indicates mean relative abundance over the respective period. Note the identical node layout in both networks. To ease comparison, also unconnected nodes are shown that feature correlations in the other network. However, unconnected nodes with zero abundance were removed, like *Pseudoramibacter* and *Aeriscardovia* in the network of stage II.

The network inferred from data of stage I only (Figure 5A) mainly consisted of two co-occurring sub-network modules. The first one was a C6/C8-related sub-network (left) characterized by positive correlations of *Lactobacillus* with unclassified *Coriobacteriaceae*, *Pseudoramibacter* with *Olsenella* and *Pseudoramibacter* with unclassified *Coriobacteriaceae*. C6 production was positively correlated with *Pseudoramibacter*, *Olsenella* and unclassified *Coriobacteriaceae*. The second sub-network (right) was acetate related. C2 production was positively correlated with *Bifidobacterium*, *Clostridiales* family XIII UCG 001, unclassified *Lachnospiraceae* and *Solobacterium*. *Lachnospira*, *Eubacterium nodatum* group and *Aeriscardovia* were also involved in positive correlations within this module.

In the network derived on data of stage II only (Figure 5B), a new C4-related sub-network module appeared. C4 production positively correlated with *Clostridium sensu stricto* 12, a genus that was positively correlated with *Ruminiclostridium* 5, unclassified *Erysipelotrichaceae* and *Bifidobacterium* here. It is worth to mention that *Atopobium* correlated with C6 and C8 positively in stage II, in which *Pseudoramibacter* had vanished.

In addition, we identified more co-occurrence pairs in a network analysis comprising stage I, transition stage and stage II (Figure 5C). Here, *Aeriscardovia* correlated positively with C6 and C8 production, while *Syntrophococcus* correlated with C4 production. Patterns of co-occurrence were detected for *Aeriscardovia* with *Pseudoramibacter* and *Syntrophococcus* with *Clostridium sensu stricto* 12, *Lachnospira*, *Solobacterium*, and *Eubacterium nodatum* group.

## Discussion

In our previous studies (Sträuber et al., 2012, 2016, 2018; Lambrecht et al., 2019), ensiled energy crops were used as substrate for MCC production. To simulate the feedstock conditions of an acidogenic fermenter fed with crop silage and producing MCCs in the present study, we selected xylan and lactate as model substrates. Feeding such defined carbon sources enabled electron balances and carbon flows. To gain further knowledge on the microbial community development in such a CE model system, we simplified the community by preventing continuous inoculation as it would have occurred in open systems.

Xylan is the major component of hemicellulose in plant cell walls (Badal, 2004). During acidogenic fermentation of corn silage, higher degradation of hemicellulose compared with cellulose was testified both in our previous batch (Sträuber et al., 2012) and continuous (Sträuber et al., 2016) studies. The xylan we used here contained more than 95% xylooligosaccharides (XOS), which is a mixture of oligosaccharides formed from xylose with a polymerization degree ranging from 2 to 10. Lactate is a typical fermentation product of LAB during the ensiling process. Previous studies have shown that the LAB *Bifidobacterium*, which was highly abundant in the inoculum of our CE process but later decreased in abundance, can ferment XOS to lactate and acetate as main products (Okazyki et al., 1990; Falck et al., 2013). *Lactobacillus*, which was highly enriched and became the most abundant LAB in our reactor, can also ferment XOS and produces mainly lactate (Kontula et al., 1998; Ananieva et al., 2012). For the other LAB we detected, such as *Aeriscardovia*, *Atopobium* and *Olsenella*, the ability to hydrolyze XOS has not been demonstrated yet. Therefore, we assume that they benefited from XOS-hydrolyzing bacteria and fermented xylose to produce mainly lactate, acetate and formate (Placidi et al., 2001; Kraatz et al., 2011). Other dominant genera, such as *Ruminiclostridium* 5, *Solobacterium*, *Syntrophococcus*, *Pseudoramibacter*, *Eubacterium nodatum* group, *Clostridium sensu stricto* 12, and *Lachnospira*, were not yet reported to hydrolyze XOS.

Our study was designed to include hydrolysis and primary fermentation in the CE process. Acetate-producing xylose fermenters such as *Syntrophococcus* (Dore and Bryant, 1990) and butyrate-producing xylose fermenters such as *Lachnospiraceae* (Cotta and Forster, 2006) and *Solobacterium* (Kageyama and Benno, 2000) can be assumed to be involved in primary fermentation. Lactate-utilizing species of the genus *Eubacterium* were reported to produce mainly butyrate (Duncan et al., 2004), which indicates that CE with lactate may also be involved in C4 production. The produced intermediates (C2 and C4) can be elongated to MCCs with lactate as electron donor. We suppose that, besides the lactate fed as substrate, *in situ* lactate formation from xylan as mentioned above contributed to the CE process. The dominant genus *Ruminiclostridium* 5 might have been the potential CE bacteria, as strain CPB6 belonging to this genus was described to catalyze CE with lactate (Zhu et al., 2017). Another genus potentially involved in MCC production is *Pseudoramibacter*, which dominated only in stage I characterized by high C6/C8 concentrations. *Candidatus* P. fermentans was predicted to use lactate as substrate for CE (Scarborough et al., 2019). MCC production directly from xylose without external electron donor, which might also have been a possible process in our reactor, was described for *Candidatus* Weimerbacter bifidus of the family *Lachnospiraceae* (Scarborough et al., 2019).

Overall, our CE process showed diverse functions including hydrolysis of XOS (e.g., by *Lactobacillus*), primary fermentation of xylose to acids (by *Syntrophococcus* for C2, *Lachnospiraceae* for C4, and *Lactobacillus* for lactate) and CE with lactate (by *Eubacterium*, *Ruminiclostridium* 5 and *Pseudoramibacter*).

To identify potential ecological interactions within microbial communities, correlation-based network analysis may help understand the guiding rules of community assembly and decipher the community dynamics (Röttjers and Faust, 2018). Process parameters were also included in the network analyses in some studies on artificial microbial ecosystems (Ju and Zhang, 2015; Ziels et al., 2018) to understand the change of process performance and further to maintain process stability. For our CE microbiota, we detected pairwise relationships among taxa, among abiotic parameters, and between taxa and parameters over time during the different process periods.

Generally, many more positive correlations were observed in stage I than in stage II. In stage I, the higher C6/C8 productivity can be explained by *Pseudoramibacter* that served as a key taxon within the C6/C8-related sub-network. In stage II, the C6/C8-related sub-network was less complex and only determined by *Atopobium*. The C4-related sub-network emerged in stage II with the key taxon *Clostridium sensu stricto* 12, explaining the higher C4 productivity.

Co-occurrences between pairs of phylogenetically distant taxa may suggest bacterial cooperation such as mutualism. As reported by Ju and Zhang (2015), the co-occurrence between ammonia-oxidizing bacteria of the genus *Nitrosomonas* and nitrite-oxidizing bacteria of the genus *Nitrospira* most likely suggests their mutualistic interactions in activated sludge. In our bioreactor, a typical example was the co-occurrence of *Pseudoramibacter* (phylum *Firmicutes*) and *Olsenella* (phylum *Actinobacteria*) in stage I (Figure 5A). Chain elongators such as *Pseudoramibacter* might use lactate released from LAB such as *Olsenella* to produce C6/C8. Lactate-based CE driven by *Olsenella* was recently reported by Lambrecht et al. (2019) for the reactor microbiota that served as inoculum for our reactor. We therefore assume that the C6/C8 sub-network was a key feature of the CE process based on corn silage, which probably persisted after adaptation to the defined carbon sources at least during stage I. The other example of cooperation was shown in the C4-related sub-network of stage II (Figure 5B). Here, *Bifidobacterium* (phylum *Actinobacteria*) co-occurred with *Clostridium sensu stricto* 12 (phylum *Firmicutes*), the latter ASV sharing a high similarity (98.3% BLAST identity) with *Clostridium luticellarii*. We propose that *Clostridium sensu stricto* 12 may have used acetate and lactate released from *Bifidobacterium* to produce C4 in our system. *C*. *luticellarii* was also assumed as the dominant candidate for performing methanol-based CE in the study of de Smit et al. (2019). Most importantly, not only lactate was provided as electron donor for CE, but also removing lactate as the reaction product shifts the reaction equilibrium toward more lactate production. In other words, LAB might increase the availability of energy from such shift. Such synergy between producer and consumer constitutes a division of labor cooperation revealed as mutual benefit (González-Cabaleiro et al., 2015).

By supplying a finite carbon resource in a CE system, it is reasonable to assume that bacterial competition also impacts unignorably the community structure, manifesting the shift of process performance. As reported in the literature, C4-producers like species of the genus *Lachnospira* (Cotta and Forster, 2006) can ferment xylose. In our CE system, the negative correlations between the functional groups of lactate producers (*Olsenella*, *Lactobacillus*) and C4 producers (e.g., *Lachnospira*) (Figure 5B) may potentially reflect the competition for the carbon and energy source xylose. This competition might direct the carbon flow more to C4 as observed in stage II. Such negative interactions between different functional groups may have some agreement with previous findings in other biotechnological systems such as wastewater treatment plants (Ju and Zhang, 2015). Likewise, under such resource-limited conditions, widespread competition between taxa crucially structures the microbial community. Finally, although integrating process parameters and absolute biomass can effectively support our hypotheses of bacterial cooperation and competition, the true ecological interactions still need to be validated in culture-dependent experiments with defined synthetic communities of species with known metabolic functions.

During long-term reactor operation, we found that the reactor microbiota self-optimized to yield more biomass at the cost of C6/C8 yields. This indicates that the C6/C8-producing bacteria in our system could not successfully compete with C4-producing bacteria during the battle for the finite resources. This might be different in open systems such as those fed with complex biomass, where new microorganisms including different CE bacteria can enter the system during operation. In our model system, the absolute abundance of potential C6-producers such as *Ruminiclostridium* 5 decreased significantly in stage II, and the genus *Pseudoramibacter* was even completely washed out after the transition period. Instead, other functional groups including potential C2-producers (e.g., *Syntrophococcus*), LAB (*Lactobacillus*) and C4-producers (*Clostridium sensu stricto* 12, *Solobacterium*, *Eubacterium nodatum* group and *Lachnospira*) increased in their absolute abundances. The negative correlations between CE bacteria of the genus *Pseudoramibacter* and other functional groups are shown in Figure 5C. Considering the higher C2 and C4 electron recoveries in stage II, we conclude that bacteria of these functional groups (i.e., C2-producers, LAB and C4-producers) captured energy from the substrate more efficiently than C6/C8-producing bacteria for their growth. Our results may confirm the theory that maximum metabolic energy harvest rate for growth can select the microbial catabolic activities in microbial ecosystems (González-Cabaleiro et al., 2015). As for the CE process, longer pathways with higher input of metabolic labor decrease the energy harvest rate. This applies to another recent study on the CE process, which also explained this effect well from the thermodynamics perspective (Wu et al., 2018). For our system, the pattern of competition would be more favored due to the following reasons. First, the enriched members share overlapping metabolic niches and require the same nutrients as so many species are functionally similar. After inoculation of the reactor, no new microorganisms with different metabolic needs or capabilities were brought into this insular community. Second, in our reactor system, all nutrients are well mixed, thus limiting spatial heterogeneity and consequently different niches, but favoring nutrient availability. In both aspects, the conditions in our system differ from those in systems with complex biomass substrate. Moreover, our resource-limited reactor drove selection for favoring bacteria that rapidly grow to take up resources (Maitra and Dill, 2015). As a consequence, community dynamics over time depends on the selection pressures mentioned above (Ghoul and Mitri, 2016). Therefore, competition cannot be avoided when using mixed cultures for producing MCCs. However, the degree of competition might be different in open systems. To what extent our observations could be extrapolated to more complex systems needs to be further tested. Until now, many studies focused mainly on other competing processes such as methanogenesis (Grootscholten et al., 2014), bacterial sulfur reduction (Cavalcante et al., 2016) and the acrylate pathway in lactate-based CE (Kucek et al., 2016) to ensure effective MCC production. Such processes were not observed here. However, the C4-producers should be also realized as a competitor for utilizing carbon sources and other nutrients. In this study, the processes of xylose fermentation to butyrate and lactate-based CE of acetate both contributed to the C4 production. In the mixed microbial fermentation study of Scarborough et al. (2018a), these were also described as the competing processes in CE for producing MCCs.

Another possible explanation is that product inhibition also promoted the community shift. With an operation at pH 5.5, the protonated C6 (0.72 g L^–1^) and C8 (0.15 g L^–1^) in stage I showed inhibitory concentrations comparable to the system reported by Andersen et al. (2017).

Furthermore, other environmental factors such as HRT and pH probably also influenced the community development and the MCC production during the long-term reactor operation. Since the operation conditions were not changed in this study, future experiments could investigate the effects of certain abiotic factors on the CE community assembly by changing them.

Our findings showed that hydrolysis, primary fermentation and CE with lactate functions were all enriched in the reactor microbiota by feeding xylan and lactate as the substrates. Ecological interactions such as cooperation between LAB and CE bacteria, as well as competition between C6/C8-producing bacteria and C4-producing bacteria, resulted in the community development over four succession stages. The higher biomass and C4 yields at the cost of C6/C8 yields may be explained by the ecological interactions discussed above. Additionally, the lower reactor performance in terms of C6/C8 production could be attributed to the loss of diversity in stage II, as the more diverse community in stage I might have a higher capacity to use redundant pathways, resulting in more efficient community functions (Werner et al., 2011). In conclusion, during the long-term reactor operation without tuning any process parameters, the CE reactor microbiota developed toward predominating C4 and biomass production instead of MCC production in our system.

## Data Availability Statement

The datasets generated for this study can be found in the Supplementary Material B and the EMBL European Nucleotide Archive (ENA) under accession number PRJEB34417 (http://www.ebi.ac.uk/ena/data/view/PRJEB34417).

## Author Contributions

BL, HS, and SK designed the study and the experiments and contributed to data analysis and data interpretation. BL performed the experiments and analyzed the reactor data as well as T-RFLP and amplicon sequencing data. FC did the network analysis. HH contributed to the discussion of the results. All authors critically contributed to the preparation of the manuscript, read and approved the final manuscript.

## Conflict of Interest

The authors declare that the research was conducted in the absence of any commercial or financial relationships that could be construed as a potential conflict of interest.
