# Supplementary material for: Competition Between Butyrate Fermenters and Chain-Elongating Bacteria Limits the Efficiency of Medium-Chain Carboxylate Production
Source: Front Microbiol. 2020 Mar 6;11:336. doi: 10.3389/fmicb.2020.00336 (PMC7067704; doi:10.3389/fmicb.2020.00336)
Supplement: Supplementary file 1 [file Data_Sheet_1.pdf]

Supplementary Material

Competition between Butyrate Fermenters and Chain-elongating Bacteria Limits the Efficiency of Medium-chain Carboxylate Production

Bin Liu, Sabine Kleinsteuber, Florian Centler, Hauke Harms, Heike Sträuber*

Department of Environmental Microbiology, Helmholtz Centre for Environmental Research – UFZ, Leipzig, Germany

*** Correspondence:**
Corresponding Author
heike.straeuber@ufz.de

Supplementary Figures and Tables

1.1 Supplementary Figures

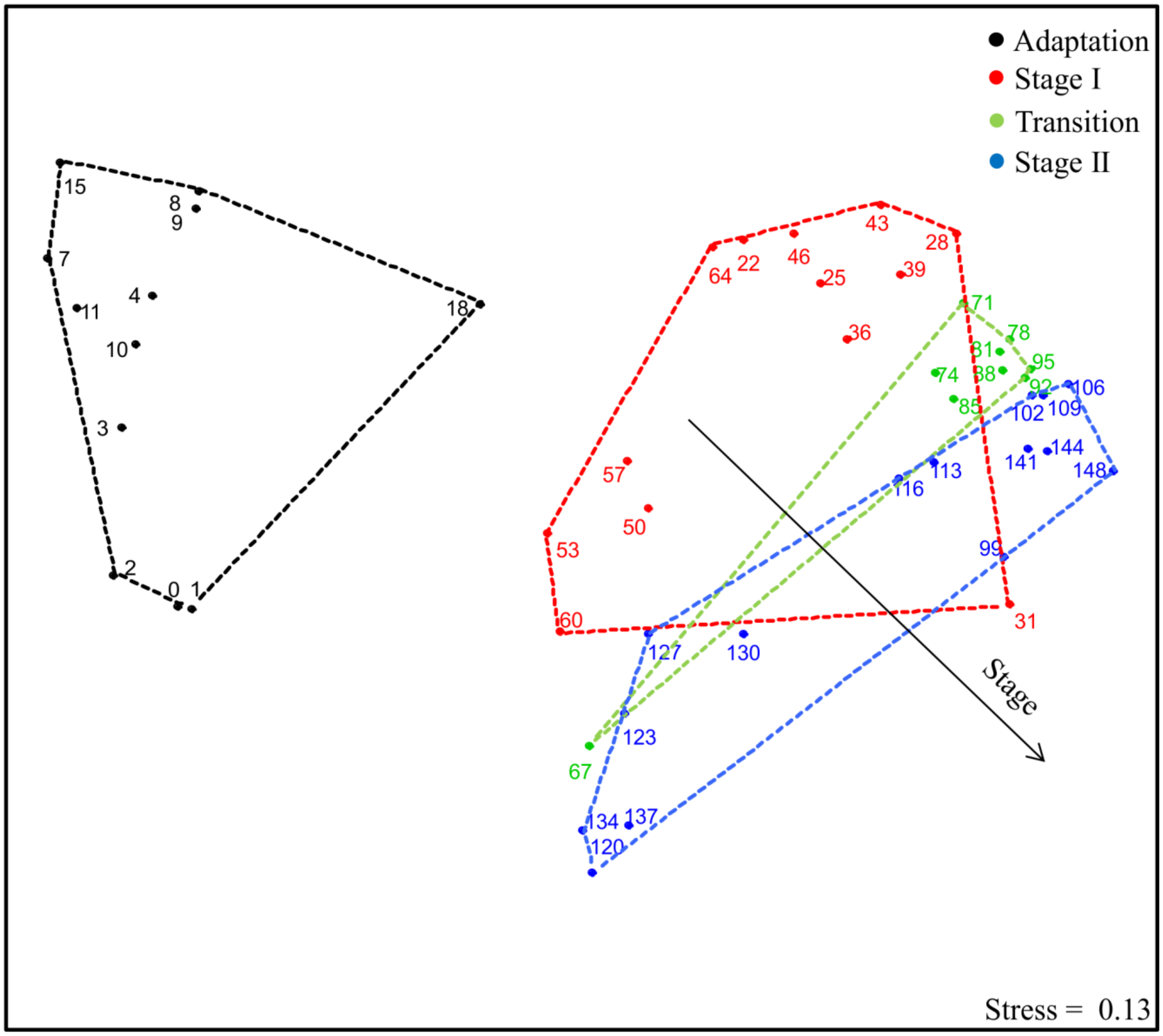

Figure S1 Bacterial community dynamics in the four succession stages, illustrated by nonmetric multidimensional scaling (NMDS) of T-RFLP profiles (16S rRNA gene amplicons, restriction enzyme *Msp*I). Data points are named according to sampling days. Proximity of data points represents community similarity based on the Bray-Curtis index. Colored polygons indicate sampling days of each succession stage. The vector shows community shifts within the temporal dynamics ($P < 0.01$, significance calculated by Monte-Carlo test with 999 permutations).

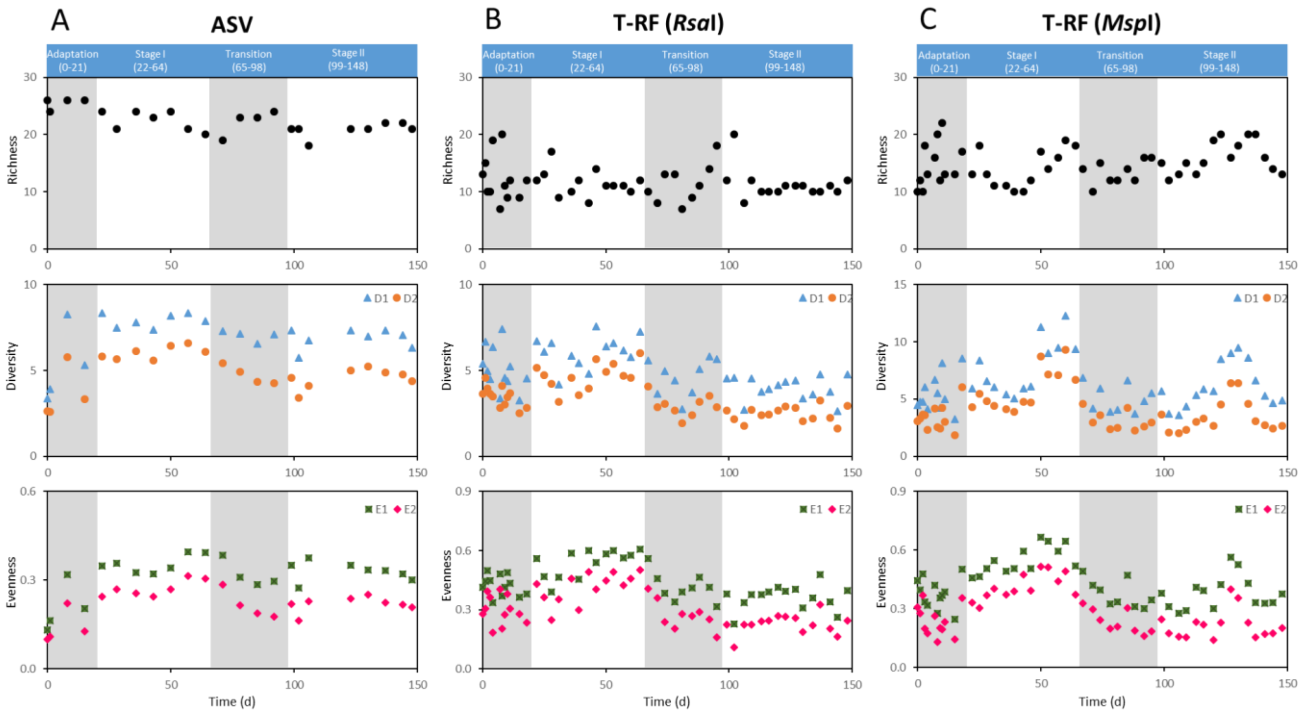

Figure S2 Alpha diversity metrics (richness, diversity and evenness) in the four succession stages based on the relative abundance of amplicon sequence variants (ASV) (**A**) and of terminal restriction fragments (T-RF) of 16S rRNA gene amplicons for restriction enzymes *Rsa*I (**B**) and *Msp*I **(C)**. D1: diversity of order one (q = 1), D2: diversity of order two (q = 2); E1: evenness of order one (q = 1), E2: evenness of order two (q = 2).

1.2 Supplementary Table

Table S1 Electron-equivalent balance in stages I and II showing mean values obtained from different sampling points in both stages, represented by 95% confidence intervals. The electron balance calculation was based on the number of electrons and the molar mass of the reduced compounds.

	Stage I		Stage II	
(mol/L)	Mean	Standard deviation	Mean	Standard deviation
Acetate[1]	0.13138	0.01259	0.16870	0.03952
iso-Butyrate[2]	0.00000	0.00000	0.00068	0.00014
n-Butyrate[3]	0.89421	0.09115	1.28598	0.11784
iso-Valerate[4]	0.00038	0.00009	0.00073	0.00018
n-Valerate[5]	0.00345	0.00026	0.01763	0.00193
n-Caproate[6]	1.00428	0.07569	0.49796	0.04068
n-Heptanoate[7]	0.00124	0.00008	0.00630	0.00028
n-Caprylate[8]	0.22191	0.01219	0.06714	0.00303
n-Nonanoate[9]	0.00011	0.00002	0.00020	0.00004
n-Decanoate[10]	0.00042	0.00010	0.00037	0.00006
Lactate[11]	0.12843	0.03005	0.19604	0.04347
Phenyl acetate[12]	0.00106	0.00147	0.00345	0.00112
Phenyl propionate[13]	0.00000	0.00000	0.00855	0.00175
Ethanol[14]	0.02892	0.00208	0.01566	0.00795
2-Butanol[15]	0.00000	0.00000	0.00017	0.00020
1-Propanol[16]	0.00152	0.00058	0.00370	0.00073
1-Butanol[17]	0.00825	0.00058	0.01835	0.00270
Biomass[18]	0.13511	0.00464	0.23645	0.01400
H_2[19]	0.01850	0.00341	0.01257	0.00584
Xylan[20]	0.04061	0.03988	0.00455	0.01016
Total Output	2.61978	0.07519	2.54884	0.12050
Total Input	2.84848	0	2.84848	0
Electron Balance	92%	3%	89%	4%

Number of electrons (mol mol^{-1}): [1]Acetate 8, [2]*iso*-Butyrate 20, [3]*n*-Butyrate 20, [4]*iso*-Valerate 26, [5]*n*-Valerate 26, [6]*n*-Caproate 32, [7]*n*-Heptanoate 38, [8]*n*-Caprylate 44, [9]*n*-Nonanoate 50, [10]*n*-Decanoate 56,

[11]Lactate 12, [12]Phenyl acetate 36, [13]Phenyl propionate 42, [14]Ethanol 12, [15]2-Butanol 24, [16]1-Propanol 18, [17]1-Butanol 24, [18]Biomass 6 × n, [19]H_2 2, [20]Xylan 20 × n; "n" stands for the degree of polymerization. The electron number of cell biomass was calculated by using a theoretical molecular composition; the empirical formula of microorganisms was assumed as $CH_{1.8}O_{0.5}N_{0.2}$ (molar mass = 24.6 g mol^{-1}). *The molar mass* (g mol^{-1}): [1]Acetate 60, [2]*iso*-Butyrate 88, [3]*n*-Butyrate 88, [4]*iso*-Valerate 102, [5]*n*-Valerate 102, [6]*n*-Caproate 116, [7]*n*-Heptanoate 130, [8]*n*-Caprylate 144, [9]*n*-Nonanoate 158, [10]*n*-Decanoate 172, [11]Lactate 90, [12]Phenyl acetate 136, [13]Phenyl propionate 150, [14]Ethanol 46, [15]2-Butanol 74, [16]1-Propanol 60, [17]1-Butanol 74, [18]Biomass 24.6 × n, [19]H_2 1/22.4, [20]Xylan 132 × n; "n" stands for the degree of polymerization.

Table S2 COD balances in stage I and stage II. By using COD kit, one point was selected per week in stage I and stage II, results are shown below. The "Input" means measured results of mineral medium with lactate and xylan added. The "Output" means measured results of effluents. COD balance was calculated from the quotient of "Output" divided by "Input". Within 95% confidence intervals, standard deviation was calculated from three measured values.

	**Time (d)**	**Input (gCOD L^{-1})**	**Standard deviation**	**Output (gCOD L^{-1})**	**Standard deviation**	**COD balance (%)**	**Standard deviation**
Stage I	36	23.48	0.31	20.37	0.14	92.0	0.7
	43	23.48	0.31	19.94	0.09	90.0	0.4
	50	23.48	0.31	20.25	0.15	91.4	0.7
	57	23.48	0.31	19.94	0.08	90.0	0.3
	64	23.48	0.31	20.38	0.17	92.0	0.8
Stage II	120	23.48	0.31	20.52	0.15	92.7	0.7
	127	23.48	0.31	20.02	0.13	90.4	0.6
	134	23.48	0.31	20.96	0.13	94.6	0.6
	141	23.48	0.31	20.38	0.08	92.0	0.3
	148	23.48	0.31	20.19	0.36	91.2	1.6
